# Characteristics of a newly diagnosed Polish cohort of patients with neurological manifestations of Wilson disease evaluated with the Unified Wilson’s Disease Rating Scale

**DOI:** 10.1186/s12883-018-1039-y

**Published:** 2018-04-05

**Authors:** Anna Członkowska, Tomasz Litwin, Karolina Dzieżyc, Michal Karliński, Johan Bring, Carl Bjartmar

**Affiliations:** 1 2nd Department of Neurology, Institute of Psychiatry and Neurology, 02 957 Warsaw, Poland; 2 Department of Experimental and Clinical Pharmacology, Medical University of Warsaw, Warsaw, Poland; 3 Statisticon AB i Uppsala, Uppsala, Sweden; 4 Wilson Therapeutics AB, Stockholm, Sweden

**Keywords:** Wilson disease, Disability evaluation, Neurological manifestations, Unified Wilson’s Disease Rating Scale (UWDRS)

## Abstract

**Background:**

Wilson disease is a rare genetic disorder in which impaired copper excretion results in toxic copper levels and tissue damage. Manifestations are primarily hepatic and/or neuropsychiatric, with a variety of neurological phenotypes. The aim of this study was to characterize neurological signs of Wilson disease in newly diagnosed patients and to determine whether they correlated with disability, liver function, and copper metabolism.

**Methods:**

Fifty-three treatment-naïve patients recently diagnosed with Wilson disease who exhibited neurological symptoms were included. Neurological manifestations were characterized by examination in terms of symptom type and degree of neurological impairment (Unified Wilson’s Disease Rating Scale [UWDRS] Part III) and correlated with degree of disability (UWDRS Part II), abnormalities in copper parameters and hepatic status.

**Results:**

Most patients (62.3%) exhibited tremor and ataxia, whereas 15.1% were dystonic, and 11.3% had parkinsonism. Discrete or unclassified signs only were observed in 11.3% of patients. A good correlation between disability (UWDRS Part II) and neurological impairment (UWDRS Part III) was observed (Pearson *r* = 0.84). However, there was a lack of correlation when either disability or neurological impairment were analyzed with copper parameters or liver impairment.

**Conclusions:**

The predominant neurological manifestations in this cohort of newly diagnosed Wilson disease patients were ataxia and tremor. Neurological impairment measured was highly correlated with the level of disability. However, hepatic manifestations of Wilson disease and copper levels did not appear to be correlated with neurological status and disability. These results highlight the challenges faced when assessing Wilson disease with its highly variable symptomatology.

**Electronic supplementary material:**

The online version of this article (10.1186/s12883-018-1039-y) contains supplementary material, which is available to authorized users.

## Background

Wilson disease (WD) is a rare autosomal recessive genetic disorder caused by mutations in the *ATP7B* gene located on chromosome 13 [1]. ATP7B is a P-type adenosine triphosphatase (ATPase) expressed mainly in hepatocytes that is involved in the transmembrane transport of copper. Decreased function of the ATP7B protein reduces the incorporation of copper into ceruloplasmin and leads to copper accumulation in the liver, brain, and other organs [2, 3].

It has previously been estimated that approximately 1 in 30,000 people worldwide have WD, with a heterozygote carrier frequency of 1 in 90. However, results from biochemical and genetic prevalence studies suggest that WD may be more common than previously estimated and the disease may be unrecognized in a substantial number of individuals [4, 5].

Typical presentation of WD is in adolescence to early adulthood, but it may occur at any age [6, 7]. Clinical presentation can vary widely in terms of type and severity, but the key features are various degrees of liver disease, neuropsychiatric manifestations and Kayser-Fleischer rings [2, 8, 9]. Although the initial signs and symptoms of WD can be classed as primarily hepatic (40%), neurological (40%), and psychiatric or asymptomatic (20%), it should be noted that patients often develop combined hepatic and neurological, or psychiatric disease [8, 9]. Untreated WD is universally fatal, commonly due to liver disease, or progressive neurological deterioration although the prognosis has improved due to available treatments [6, 9].

The neurological abnormalities of WD show marked variation in both type of presentation and severity, but can generally be classified into syndrome types based on predominant symptoms, such as tremor and ataxia, bradykinesia (parkinsonism-like), and dystonia. In many cases, classification of neurological features is challenging as patients can have various signs and more than one abnormality, each with a different level of severity [8, 9].

The Unified Wilson’s Disease Rating Scale (UWDRS) was developed as a tool for the comprehensive evaluation of neurological signs and symptoms in patients with WD. The scale is based on partly modified elements from well-established and validated scales assessing neurological status, such as the Barthel index, the Unified Parkinson’s Disease Rating Scale (UPDRS), and the Unified Huntington’s Disease Rating Scale (UHDRS) [8, 10]. Part I of UWDRS concerns consciousness, while Part II evaluates disability. UWDRS Part III involves a neurological examination and uses clinical rating scales e.g., for tremor and cerebellar disorders to measure neurological signs [8, 10].

Data regarding correlation of the neurological phenotype with disability, hepatic status, as well as copper metabolism, are sparse [11–13]. In the current study, we analyzed a cohort of newly diagnosed and previously untreated WD patients with neurological symptoms who were consecutively admitted to our center. The aim was to characterize the neurological manifestations in terms of symptom type and degree of neurological impairment (UWDRS Part III) and correlate these features with degree of disability (UWDRS Part II), abnormalities in copper metabolism, and hepatic status.

## Methods

The study was conducted at a single center, the 2nd Department of Neurology, Institute of Psychiatry and Neurology, Warsaw, Poland. Treatment naïve patients were consecutively enrolled as they were seen at the hospital over a period from 2005 to 2014. Neurologically asymptomatic patients with WD who presented with hepatic, psychiatric or preclinical forms of the disease, or patients previously receiving any WD therapy, were excluded. All patients were diagnosed with WD based on a combination of abnormal copper results, presence of a Kayser-Fleisher ring and genetic testing results, performed in our center, as previously described [14, 15]. Ethics committee approval was obtained from the local bioethics committee and all patients provided written informed consent prior to participation.

### Neurological assessments

The main outcome variable of the study was the UWDRS Parts II and III. The UWDRS consists of three parts including consciousness (Part I, single item), activities of daily living (based on the Barthel Scale) as reported by the patient or their proxy and verified by the neurologist if possible (Part II, items 2–11), and a detailed neurological examination (Part III, items 12–34) [8, 10].

In addition, patients were further classified based on the predominant neurological syndrome type at diagnosis by experienced neurologists using best clinical judgement and based on classifications described by Marsden [16] and Oder et al. [17], i.e., tremor (including patients with predominant tremor and ataxia), parkinsonism (including rigidity, rest tremor and hypokinesia), dystonia (including choreoathetosis), or discrete neurological/unclassified signs not encompassed by these classifications, such as slight dysarthria, drooling, hypomimia, occasional mild tremor etc.

### Copper measurements and other assessments

Serum ceruloplasmin was measured using the improved colorimetric enzymatic assay developed by Ravin in 1961 [18]. Total serum copper concentration and 24-h urinary copper excretion were measured by flame atomic absorption spectrometry. The levels of free copper, measured as non-ceruloplasmin bound copper (NCC), were calculated according to previously described formula [19–21].

Basic laboratory liver tests (i.e., enzymatic, synthetic, and bilirubin) were performed in the hospital laboratory using standard methods.

### Statistical analysis

Categorical variables are presented as a number of valid observations and proportions calculated with exclusion of unknown values from the denominator. Continuous variables are presented as a mean with standard deviation (SD).

Pearson’s correlation coefficient (*r*) was used to express correlations between UWDRS Part II score and UWDRS Part III score in the whole cohort and according to predominant clinical manifestation. Additionally, similar correlations were calculated between UWDRS score and baseline copper levels, hepatic parameters, age at onset of symptoms and age at the diagnosis. Calculations were carried out using STATISTICA 12.0 software package (Stat Soft Inc., Tulsa, USA, 2013). *P*-values < 0.05 were considered statistically significant.

## Results

A total of 53 newly diagnosed WD patients (30 males, 23 females) with neurological manifestations were included in the analysis (Table 1). Most patients had liver test results that were in the normal range or only slightly/moderately elevated as shown in Table 1. In the majority of patients, compared with the normal range, ceruloplasmin, and total serum copper concentrations were low (mean 13.7 mg/dL and 62.0 mg/dL, respectively), whilst urinary copper excretion was elevated (mean 128 μg/dL/24 h) (Table 1). Mean NCC levels were elevated (3.4 μmol/L; SD 2.0, range 0.6 to 10.9).

**Table 1:** Characteristics and laboratory values at baseline

	*n*	Study population Mean (SD) unless otherwise stated	Normal range
Female gender (*n* [%])	53	23 (43.4)	
Age (years)	53	36.2 (11.3)	
Age at onset (years)	52	30.4 (12.5)	
Onset to diagnosis (years)	52	5.6 (6.6)	
Presence of Kayser-Fleischer ring (*n* [%])	53	44 (83.0)	
Ceruloplasmin (mg/dL)	53	13.7 (6.3)	25–45
Serum copper (μg/dL)	53	62.0 (17.8)	70–140
Urinary copper (μg/24 h)	53	128 (142)	< 50
Non-ceruloplasmin-bound copper (μmol/L)	53	3.4 (2.0)	^a^
Alanine aminotransferase (IU/L)	53	38 (43)	< 41
Aspartate aminotransferase (IU/L)	53	36 (21)	< 40
Gamma-glutamyltransferase (IU/L)	52	70 (57)	< 42
International normalized ratio	52	1.3 (0.2)	0.8–1.2
Bilirubin (mg/dL)	53	1.1 (0.6)	< 1
Serum albumin (g/dL)	47	4.0 (0.7)	3.5–5.0

*SD* standard deviation

^a^Non-ceruloplasmin-bound copper (NCC) is not commonly used in clinical practice in healthy individuals as approximately 80% have negative values. NCC has been reported to be above 4 μmol/L in most patients with Wilson disease [21]

The most common individual neurological signs were dysarthria (73.6%), arms postural tremor (69.8–71.7%), impaired finger tapping (66.0%), impaired posture (66.0%), and reduced facial expression (66.0%) (Table 2). The syndrome of highest prevalence was ataxia/tremor (62.3%) followed by dystonia (15.1%) and parkinsonism (11.3%). A small proportion of patients (11.3%) had only discrete signs or were considered as unclassified.

**Table 2:** Most common neurological signs and symptoms (present in > 50% of patients)

Symptom	*n*	Any abnormality, %	Mean score in UWDRS item
Speech impairment (dysarthria)	53	73.6	1.17
Postural tremor in left arm	53	71.7	1.11
Postural tremor in right arm	53	69.8	1.23
Impaired left finger tapping	53	66.0	1.11
Impaired right finger tapping	53	66.0	1.13
Impaired posture	53	66.0	0.66
Reduced facial expression (hypomimia)	50	66.0	1.02
Impaired handwriting	51	56.9	1.24
Impaired rapid alternating movements of left hand	53	54.7	0.92
Impaired rapid alternating movements of right hand	53	50.9	0.89

*UWDRS* United Wilson’s Disease Rating Scale

**Fig. 1:**
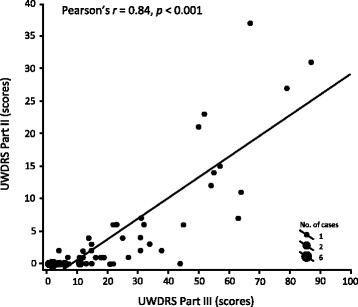
Correlation between UWDRS Part II and UWDRS Part III Score (total cohort). *UWDRS* United Wilson’s Disease Rating Scale

Mean Part II UWDRS score was 5.0 (SD 8.5, range 0 to 37) and mean Part III UWDRS score was 24.4 (SD 22.3, range 1 to 87) (Table 3). Overall individual scores in UWDRS Part III (neurological signs) were found to strongly correlate with Part II individual scores (disease-related disability; *r* = 0.84, *p* < 0.001) (Fig. 1, Table 3). In addition, there was a strong correlation between Part III and Part II scores in subgroups according to the predominant neurological syndrome, ataxia/tremor, dystonia, or parkinsonism (Table 3).

**Table 3:** Correlations between UWDRS Part II and UWDRS Part III scores according to predominant clinical manifestation of Wilson disease

Predominant clinical manifestation	UWDRS II score Mean (SD) [range]	UWDRS III score Mean (SD) [range]	Pearson’s correlation coefficient, *r* (*p*-value)
Overall (*n* = 53)	5.0 (8.5) [0–37]	24.4 (22.3) [1–87]	0.84 (*p* < 0.001)
Ataxia/tremor (*n* = 33)	3.8 (6.4) [0–27]	23.5 (21.1) [1–79]	0.81 (*p* < 0.001)
Dystonia (*n* = 8)	11.1 (12.7) [1–37]	36.6 (18.5) [15–67]	0.90 (*p* = 0.002)
Parkinsonism (*n* = 6)	7.8 (12.6) [0–31]	34.0 (30.8) [6–87]	0.97 (*p* = 0.001)
Discrete signs or unclassified (*n* = 6)	0.3 (0.8) [0–2]	3.7 (3.7) [0–11]	0.04 (*p* = 0.934)

*SD* standard deviation, *UWDRS* United Wilson’s Disease Rating Scale

When we evaluated correlations with laboratory parameters, there was no significant correlation between UWDRS Part II scores or UWDRS Part III scores and the serum levels of ceruloplasmin, copper, NCC, or any of the liver tests (Additional file 1: Table S1). However, UWDRS Part III scores showed a weak but statistically significant positive correlation with age at onset of symptoms (*r* = 0.34, *p* = 0.014) and age at diagnosis (*r* = 0.28, *p* = 0.039) (Additional file 1: Table S1).

## Discussion

In our study of 53 newly diagnosed and previously untreated patients with WD, most (62.3%) were classified as having primary ataxia/tremor syndrome, which generally concurs with the available literature [10, 17, 22], although comparisons between studies of neurological signs and levels of disability in WD are challenging due to the complexity of the clinical manifestations.

There are numerous scales available for parkinsonism, dystonia and ataxia, but there has been a lack of specific instruments for measuring the severity of impairment and disability associated with WD [8]. A specific rating scale for WD must be able to capture its multi-systemic impairment, be easily administered, yet be sensitive to small clinical change. There has been a reliance on the use of proxy scales, such as the Hoehn and Yahr scale, the Unified Parkinson’s Disease Rating Scale (UPDRS), the Unified Huntington’s Disease Rating Scale (UHDRS), the International Co-Operative Ataxia Rating Scale (ICARS) and the Rating Scale for Dystonia (RSD). However, these scales are not capable of capturing the distinctive and complex multi-systemic spectrum of WD and tend to include irrelevant features [23]. Also, focusing on tremor, ataxia, parkinsonism, or dystonia in isolation is insufficient to accurately reflect the neurological impairment experienced by patients with WD [10, 23]. For these reasons, Członkowska et al. developed the UWDRS [8]. It is a novel rating scale specific for WD, which was designed to accurately document the neurological and functional impairment suffered by patients with WD [8]. The UWDRS was found to capture the complex impairment seen in patients with WD and was widely used by the EuroWilson consortium and GeNeMove [8, 10] and has been used in a multicenter clinical trial in WD [24].

The current study demonstrated a significant correlation between disease-related disability (UWDRS Part II) and objective neurological examination (UWDRS Part III). This is consistent with the findings by Oder et al. demonstrating a clear-cut correlation between the severity of neurological impairment and the restriction in functional capacity [25]. In addition, Volpert et al. recently published a study demonstrating a strong correlation between “minimal UWDRS” (UWDRS Part II without one item) and total as well as neurological UWDRS scores [26]. In our study, we also demonstrated the correlation separately in all three major WD neurological subtypes. There was no correlation in subtype with discrete or unclassified signs, but the group was small and UWDRS scores by definition were low.

The management of patients with neurological WD is associated with therapeutic challenges, particularly for patients with tremor, parkinsonism, and dystonia, which are key factors contributing to the UWDRS score [10, 27]. Recommended initial pharmacological treatment for WD comprises chelating agents such as D-penicillamine or trientine, and zinc salts that reduce intestinal copper absorption. Despite these therapies, a considerable proportion of patients still experience neurological symptoms (50.5% [*n* = 106]) [28]. In particular, dysarthria/dysphagia, bradykinesia and postural tremor persist, highlighting the need for improved therapies for patients with WD [10].

To evaluate the efficacy of available WD therapies, more knowledge is needed regarding the natural course of WD, neurological features and liver impairment, as well as changes in copper parameters. The UWDRS has been shown to correlate with exchangeable copper (CuEXC) [13] and optical coherence tomography of the retina in WD patients [29], and it seems to be a useful tool to follow patients with neurological symptoms in WD. We also used this scale to evaluate treatment effects of D-penicillamine compared with zinc in our center [14].

In the current study, patients who were relatively older at diagnosis had more severe neurological impairment but without significant impact on disability. It is probable that the mild disability, even in the presence of neurological signs, led to a delay in diagnosis on the part of both the patient and physician. We found a lack of correlation between hepatic status at diagnosis and neurological impairment. Patients with acute and severe liver symptoms are usually managed by hepatology departments, and patients are generally referred to neurologists when they present with mild hepatic disease. However, most patients have some evidence of liver injury consistent with previous data [30]. Many patients who develop neurological signs as the first WD manifestation have compensated liver cirrhosis that develops insidiously [1, 2, 30].

In the current study, ceruloplasmin, urinary copper excretion, NCC, and total copper did not correlate with severity of neurological symptoms. This is consistent with previous data [11, 31], but in contrast to the results of Poujois et al. who found a correlation between UWDRS score at diagnosis and determination of CuEXC [13]. A single NCC value largely represents a snapshot of copper levels at the time of sampling, whereas the UWDRS score also represents accumulated long-term neurological manifestations resulting from chronically elevated free copper levels. Therefore, a strict correlation between these two parameters may not necessarily be expected. Regarding NCC determination, there are known differences between laboratories using different methods for measuring ceruloplasmin [32, 33]; however, all measurements of ceruloplasmin and copper in our patients were performed in the same laboratory. We found that NCC was detectable in all our patients and was in a range reported in the literature as typical for WD [21].

There were some limitations associated with our study. The analysis is based on a relatively small group of 53 patients. However, it is important to note that WD is a rare disease and the recruitment of treatment-naïve patients is challenging, even in a nationwide referral center. Many of our patients are referred from other hospitals early after treatment initiation to confirm the diagnosis and they remain under long-term outpatient care. Due to the rapid effect of D-penicillamine on copper metabolism, we decided not to include these previously treated patients in this study, which limited our sample size. Furthermore, the sample size did not allow meaningful analyses for all subgroups.

## Conclusions

The results of this study confirm a strong correlation between the degree of neurological impairment and the level of disability captured with the UWDRS. However, there was no clear association between neurological status and serum markers of hepatic status, or copper parameters. These results confirm the highly heterogeneous symptomatology of WD and emphasize the challenges of applying single tools only for assessment of patients with WD.

## Additional file

Additional file 1: **Table S1.** Correlations between UWDRS Part II and UWDRS Part III and other variables. (DOC 72 kb)

## Abbreviations

CuEXC: Exchangeable copper
ICARS: International Co-Operative Ataxia Rating Scale
NCC: Non-ceruloplasmin bound copper
RSD: Rating Scale for Dystonia
SD: Standard deviation
UHDRS: Unified Huntington’s Disease Rating Scale
UPDRS: Unified Parkinson’s Disease Rating Scale
UWDRS: Unified Wilson’s Disease Rating Scale
WD: Wilson disease
