# Supplementary material for: Characteristics of a newly diagnosed Polish cohort of patients with neurological manifestations of Wilson disease evaluated with the Unified Wilson’s Disease Rating Scale
Source: BMC Neurol. 2018 Apr 5;18:34. doi: 10.1186/s12883-018-1039-y (PMC5887239; doi:10.1186/s12883-018-1039-y)
Supplement: Supplementary file 1 — Table S1. Correlations between UWDRS Part II and UWDRS Part III and other variables. (DOC 72 kb) [file 12883_2018_1039_MOESM1_ESM.doc]

**Additional file 1: Table S1**

Correlations between UWDRS Part II and UWDRS Part III and other variables

|  | UWDRS Part II | | UWDRS Part III | |
| --- | --- | --- | --- | --- |
|  | Pearson’s correlation coefficient, *r* | *p*-value | Pearson’s correlation coefficient, *r* | *p*-value |
| Age at onset | 0.10 | 0.502 | 0.34 | 0.014 |
| Age at diagnosis | 0.05 | 0.717 | 0.28 | 0.039 |
| Years from onset to diagnosis | –0.07 | 0.599 | -0.12 | 0.382 |
| Ceruloplasmin | –0.07 | 0.598 | 0.02 | 0.873 |
| Serum copper | –0.02 | 0.888 | 0.04 | 0.789 |
| Urinary copper | –0.18 | 0.205 | –0.27 | 0.053 |
| Non-ceruloplasmin-bound copper | 0.09 | 0.522 | 0.02 | 0.899 |
| Alanine aminotransferase | –0.10 | 0.468 | –0.25 | 0.072 |
| Aspartate aminotransferase | –0.17 | 0.224 | –0.23 | 0.105 |
| Gamma-glutamyltransferase | 0.20 | 0.152 | 0.17 | 0.234 |
| International normalized ratio | 0.15 | 0.291 | 0.08 | 0.563 |
| Bilirubin | –0.07 | 0.629 | –0.09 | 0.526 |
| Serum albumin | –0.24 | 0.100 | –0.23 | 0.115 |

*UWDRS*, United Wilson’s Disease Rating Scale.
